# Obituary Notice

**Published:** 1848

**Authors:** 


### ARTICLE VII.

### OBITUARY NOTICE.

DIED.---On Wednesday, June 21st., at Algonquin, McHenry Co., Ill., of typhoid fever supervening neuralgia, *David Burton, M.D.*, aged 31 years.

Dr. Burton was a young man of superior talents and unexceptionable moral character, standing high in the estimation of his neighboring *Physicians*, and an honor to the *Profession.*

In him the *Public* deeply deplores the loss of one of its most valuable *citizens*, as well as a judicious and successful physician.---*Com.*

